# The Role of Long Noncoding RNAs (lncRNAs) in Esophageal Cancer Therapy Resistance and Metastasis

**DOI:** 10.3390/biomedicines12030660

**Published:** 2024-03-15

**Authors:** Zong-Ping Weng, Shen-Kai Hsu, Hui-Min David Wang, Kuo-Jen Chen, Po-Yen Lee, Chien-Chih Chiu, Kai-Chun Cheng

**Affiliations:** 1 School of Medicine, College of Medicine, Kaohsiung Medical University, Kaohsiung 807, Taiwan; u109001029@gap.kmu.edu.tw (Z.-P.W.); maco69@gmail.com (P.-Y.L.); 2 Department of Biotechnology, Kaohsiung Medical University, Kaohsiung 807, Taiwan; b043100050@gmail.com (S.-K.H.); cchiu@kmu.edu.tw (C.-C.C.); 3 Graduate Institute of Biomedical Engineering, National Chung Hsing University, Taichung 402, Taiwan; davidw@dragon.nchu.edu.tw; 4 Department of Ophthalmology, Kaohsiung Municipal Siaogang Hospital, Kaohsiung 812, Taiwan; 0870649@kmuh.org.tw; 5 Department of Ophthalmology, Kaohsiung Medical University Hospital, Kaohsiung 807, Taiwan

**Keywords:** esophageal cancer (EC), long noncoding RNAs (lncRNAs), drug resistance, metastasis

## Abstract

Esophageal cancer (EC) is one of the most aggressive gastrointestinal cancers. Despite improvements in therapies, the survival rate of patients with EC remains low. Metastasis accounts for up to 90% of cancer-related deaths, and resistance to anti-neoplastic therapeutics is also a main cause of poor survival. Thus, metastasis and drug resistance are undoubtedly the two main challenges in cancer treatment. Among the different categories of noncoding RNAs, lncRNAs have historically drawn less attention. However, lncRNAs have gradually become a research hotspot, and increasing research has demonstrated that lncRNAs participate in the tumorigenesis of multiple types of cancer, including EC. Long noncoding RNAs (lncRNAs) are RNA transcripts longer than 200 nucleotides in length that play important roles in epigenetics, transcription regulation, and posttranscriptional processing. In this review, we elucidated the role of lncRNAs in the metastasis and drug resistance of EC and discussed their potential clinical applications and related limitations. With a better understanding of the underlying mechanisms of lncRNAs, we can identify therapeutic targets for EC in the future.

## 1. Introduction

Esophageal cancer (EC) is one of the most aggressive malignancies. In 2020, EC ranked seventh in terms of incidence and sixth in terms of mortality among malignant tumors [1]. There are two primary forms of EC—esophageal squamous-cell carcinoma (ESCC) and esophageal adenocarcinoma (EAC)—with the former type accounting for 90% of all EC cases worldwide; however, the incidence of the latter has significantly increased in the past few decades. However, the incidence of this disease varies with geographical location [2]. The pathogenesis of EC is complex; Barret’s esophagus is the precursor for EAC, while ESCC can be caused by multiple factors (e.g., tobacco smoking, alcohol consumption, and genetic factors) [3,4]. Currently, neoadjuvant chemoradiation therapy is a key treatment for EAC patients with locally advanced and/or lymph-node-positive tumors, and postoperative adjuvant therapy has been found to increase the disease-free survival rate of ESCC patients [5]. Despite improvements in therapies, the prognosis of patients with EC remains poor, with a global 5-year survival rate of approximately 20.5% [6]. Thus, it is essential to clarify the molecular mechanism of tumor progression, which might reveal ideas for the treatment of EC. Metastasis contributes to as many as 90% of cancer-associated deaths [7], and resistance to anti-neoplastic therapeutics is another cause of poor survival because recurrence and metastasis occur after drug resistance develops [8]. Therefore, gaining a deeper understanding of the key regulators involved in these two processes might facilitate the development of better therapeutic regimens and improve the poor prognosis of patients with EC. Previous research has focused mainly on the role of protein-coding RNAs in the progression of EC, which has led to the development of numerous promising approaches for treating this disease. However, with recent advances in genomic research, accumulating studies have revealed that noncoding RNAs are also involved in various physiological and pathological processes, including tumor progression. Among the different categories of noncoding RNAs, long noncoding RNAs (lncRNAs) have drawn less attention. Nevertheless, lncRNAs have gradually become a research hotspot because they have recently been found to govern the development of multiple types of cancer, such as prostate cancer, breast cancer and EC [9]. This review highlights the roles of lncRNAs in the metastasis and drug resistance of EC.

## 2. Overview of lncRNAs

In addition to protein-coding mRNAs, human genomes also contain several other types of RNAs. According to the Human Genome Project, protein-coding genes account for less than 2% of the whole genome sequence, and many noncoding regulatory regions are transcribed into ncRNAs. ncRNAs can be divided into several categories, including ‘housekeeping’ ncRNAs (such as rRNA, tRNA, snRNA and snoRNA), regulatory ncRNAs and some other poorly characterized types of ncRNAs [10,11]. LncRNAs are regulatory ncRNAs, and their significant roles in complex organisms have recently received increased attention.

LncRNAs are RNA transcripts longer than 200 nucleotides that are poorly conserved and lack an open reading frame. Like coding genes, lncRNAs are transcribed via RNA polymerase II transcription, which includes 5′-capping, poly(A) tails and splicing through spliceosomes [12]. Based on their genomic origin, lncRNAs can be classified into five categories: sense, antisense, bidirectional, intronic and intergenic lncRNAs [13]. Although lncRNAs cannot be translated into functional proteins, they are associated with diverse biological processes. According to previous studies, lncRNAs can participate in the epigenetic regulation of gene expression by recruiting chromatin-modifying proteins (e.g., methyltransferases, acetyltransferases, and deacetylases) to specific genomic loci through cis-regulation or trans-regulation [14]. For instance, the lncRNA Hox transcript antisense RNA (HOTAIR) regulates the expression of HOXD genes by recruiting polycomb chromatin remodeling complex 2 (PRC2) [15]. In addition, lncRNAs can participate in transcriptional regulation by influencing the activity of specific transcription factors and polymerases that regulate gene transcription. Moreover, lncRNAs can affect posttranscriptional processing steps, such as splicing, transport, translation and degradation, by interacting with mRNAs. For instance, they can serve as ‘‘miRNA sponges’’ that modulate the competing endogenous RNA (ceRNA) network [16]. As lncRNAs participate in a variety of mechanisms that regulate gene expression, abnormal expression of lncRNAs can result in human diseases such as cardiovascular disease and multiple types of cancer development, including EC [17,18]. In the section below, we summarize the roles of lncRNAs in the development of EC.

## 3. LncRNAs Participate in the Process of EC Metastasis

**Figure 1:**
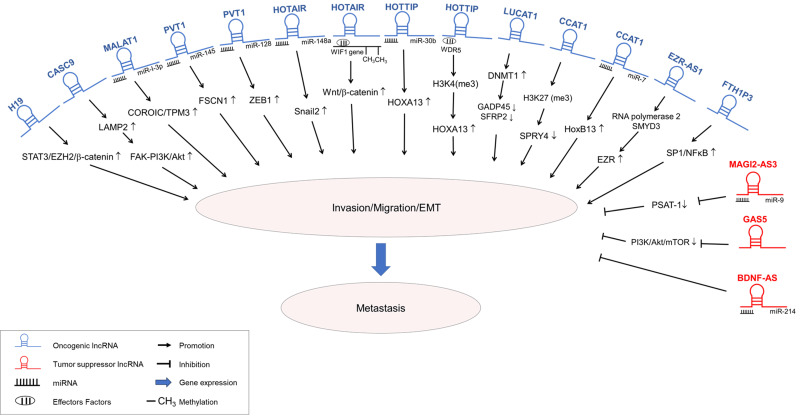
Long noncoding RNA (lncRNA)-controlled pathways in the metastasis of esophageal carcinoma cells (ECC). These pathways involve migration, epithelial–mesenchymal transition (EMT), and invasion, which lead to the metastasis of ECC. Two distinct types of lncRNAs are depicted: oncogenic lncRNAs (blue) promote metastasis, while tumor suppressor lncRNAs (red) attenuate metastasis of ECC.

Metastasis is a sequential and multistep process during which malignant cells spread from the primary tumor to distant organs; it can be divided into five major steps: invasion, intravasation, dissemination, extravasation and colonization [19,20]. Accumulating research has suggested that lncRNAs can mediate EC metastasis through diverse mechanisms. LncRNAs have dual roles in metastasis: (1) Some lncRNAs function as promotors of metastasis, and (2) others function as repressors of metastasis; the detailed regulatory mechanisms of these two types of lncRNAs are discussed in the following section (Figure 1 and Table 1).

### 3.1. LncRNAs That Function as EC Metastasis Promoters

#### 3.1.1. H19

H19 has been proven to play crucial roles in multiple types of cancer since it was first discovered by Bartolomei and colleagues in 1991 [21,22,23,24]. H19 expression is upregulated in human EC tissues and cell lines compared to that in corresponding normal controls. In addition to promoting the proliferation of EC cells by regulating cell cycle progression at the G0/G1 phase [25,26], H19 contributes to invasion and epithelial-to-mesenchymal transition (EMT), as its knockdown in ESCC cells leads to the upregulation of epithelial markers, e.g., E-cadherin, and the downregulation of mesenchymal markers, e.g., vimentin and MMP-9 [26]. Regarding the possible mechanism of EMT, Chen and associates demonstrated that high expression of H19 results in increased expression of signal transducer and activator of transcription 3 (STAT3) and Drosophila zeste gene enhancer human homolog 2 (EZH2), and they cooperatively promote EMT and metastasis via the SRY-box transcription factor 4 (SOX4)-β-catenin pathway [27].

#### 3.1.2. CASC9

The lncRNA cancer susceptibility candidate 9 (CASC9) is another important player that promotes EC metastasis, and its expression is significantly greater in ESCC cells and tissues than in normal controls. Pan and associates demonstrated that the knockdown of CASC9 suppressed the migration and invasion of ESCC cells [28]. Additionally, CASC9 was shown to promote EMT, which was accompanied by the upregulation of EMT-related markers [29]. Liang et al. further reported that by binding to CREB-binding protein (CBP), CASC9 can recruit CBP to the promoter of laminin subunit gamma-2 (LAMC2), subsequently activating the focal adhesion kinase (FAK)-phosphoinositide-3-kinase (PI3K)/protein kinase B (Akt) pathway to promote ESCC metastasis [30].

#### 3.1.3. MALAT1

The lncRNA metastasis associated with lung adenocarcinoma transcript-1 (MALAT1) is also involved in the invasion and metastasis of EC. Aberrant expression of MALAT1 is associated with metastasis in multiple types of cancers, and MALAT1 can act as a metastasis promoter or metastasis suppressor depending on the cancer type. For example, MALAT1 facilitates metastasis in colon cancer [31], gastric cancer [32] and ovarian cancer [33] but suppresses metastasis in breast cancer [34]. In EC specimens, the expression of MALAT1 is upregulated, and increased viability and migratory capacities were observed in EC cells compared to control cells. The inhibition of MALAT1 upregulated the expression of miR-1-3p via a ceRNA mechanism, which subsequently suppressed tumor cell metastasis and tumor angiogenesis by inactivating the downstream effectors Coronin-1C (CORO1C) and tropomyosin 3 (TPM3). Downregulation of CORO1C/TPM3 signaling also contributed to the inhibition of EC tumor metastasis in vivo [35]. Chen et al. demonstrated that silencing MALAT1 resulted in altered expression of EMT-related markers. However, overexpression of EZH2 reversed these effects. These results indicated that the Ezh2/Notch1 pathway is involved in EMT in EC cells [36].

#### 3.1.4. PVT1

Recently, several studies have shown that plasmacytoma variant translocation 1 (PVT1) is overexpressed in human tumors, including non-small cell lung cancer (NSCLC) [37], small cell lung cancer (SCLC) [38], gastric cancer [39] and breast cancer [40], where it functions as an oncogene and regulates cancer cell metastasis. Zheng et al. demonstrated that PVT1 expression was markedly greater in EC tissues than in adjacent normal tissues and promoted invasion by regulating EMT-related protein expression [41]. Shen and associates indicated that PVT1 promotes EC cell metastasis by specifically binding to miR-145. Fascin actin bundling protein 1 (FSCN1), which was previously found to promote NSCLC cell growth, migration, and invasion [42], is targeted by miR-145. Thus, PVT1 represses the expression of miR-145 to induce cell migration and invasion via the upregulation of FSCN1 [43]. Moreover, by acting as a molecular sponge, PVT1 has been demonstrated to compete with zinc finger E-box binding homeobox 1 (ZEB1) for binding to miR-128. ZEB1 was identified as a transcriptional repressor of E-cadherin due to its ability to bind to its promoter. PVT1 can upregulate the expression of ZEB1 to promote ESCC cell migration and invasion [44].

#### 3.1.5. HOTAIR

Chen and associates demonstrated that the lncRNA HOX transcript antisense RNA (HOTAIR) is upregulated in EC cells, and the invasion and migration of these cells were suppressed by transfection of KYSE30 cells with HOTAIR [45]. Xu and Zhang revealed the mechanisms by which HOTAIR promotes invasion, migration and EMT in EC. The lncRNA HOTAIR not only acts as a miRNA sponge to reduce miR-148a expression, subsequently upregulating Snail 2, but also serves as an epigenetic regulator to promote ESCC metastasis [46]. It has been reported that HOTAIR causes H3K27 trimethylation in the Wnt inhibitory factor 1 (WIF-1) promoter and inhibits its expression. With the downregulation of WIF-1, the Wnt/β-catenin pathway is activated, thereby promoting tumor migration and invasion [47].

#### 3.1.6. HOTTIP

The lncRNA HOXA transcript at the distal tip (HOTTIP) is transcribed from a genomic region at the 5′ tip of the HOXA locus containing 3764 nucleotides [48]. The expression of HOTTIP and the HOXA gene HOXA13 is upregulated in ESCC tissues and cell lines. Both of these factors induce cell migration, invasion and EMT pathway activation. Lin et al. studied the mechanism of the HOTTIP/HOXA13 interaction and showed that the lncRNA HOTTIP can recruit WD repeat domain 5 (WDR5) to the HOXA13 promoter locus, which promotes histone H3 lysine 4 trimethylation (H3K4me3) and the binding of polymerase II to the HOXA13 promoter, thus resulting in the activation of HOXA13 gene transcription. In addition, HOTTIP induces HOXA13 expression via miR-30b and Argonaute RISC catalytic component 2 (Ago2), which serve as essential components of the RNA-induced silencing complex (RISC) and as mediators of RNAi mechanisms. Hence, HOTTIP can significantly decrease miR-30b levels and subsequently downregulate the miR-30b target HOXA13 via Ago2 [49].

#### 3.1.7. LUCAT1

Accumulating evidence has demonstrated that lung-cancer-associated transcript 1 (LUCAT1), also known as smoke-and-cancer-associated lncRNA 1 (SCL1), is involved in the progression of multiple types of cancer [50,51,52,53,54]. Yoon and colleagues demonstrated that LUCAT1 expression is also increased in ESCC cell lines and cancer tissues. KYSE-30 cells and HCE-4 cells transfected with LUCAT1 siRNAs exhibited inhibited ESCC cell migration and invasion. Furthermore, they reported that LUCAT1 knockdown suppressed DNA methyltransferase 1 (DNMT1) protein expression by inducing DNMT1 ubiquitination through ubiquitin-like proteins, which include PHD and RING finger domain protein 1 (UHRF1). UHRF1 was previously found to trigger ubiquitination and thus lead to proteasomal degradation of DNMT1 [55]. Taken together, these findings indicate that LUCAT1 can activate DNMT1 to inhibit the expression of tumor suppressor genes such as GADD45G and SFRP2, facilitating ESCC cell invasion and migration and disease progression [56].

#### 3.1.8. CCAT1

Colon-cancer-associated transcript-1 (CCAT1), located at chromosomal region 8q24.21, is significantly upregulated in multiple types of cancer [57,58,59,60,61] and is also highly expressed in ESCC. Zhang et al. demonstrated that knockdown of CCAT1 significantly suppressed cell migration in vivo and in vitro. In contrast, overexpression of CCAT1 promoted cell migration in ESCC. Mechanistically, in the nucleus, CCAT1 can act as a scaffold for two distinct histone methylation complexes, leading to the repression of sprouty RTK signaling antagonist 4 (SPRY4), which is considered a tumor suppressor gene. Moreover, in the cytoplasm, CCAT1 competes for the binding of miR-7, thereby mediating posttranscriptional regulation of HOXB13. Both of these mechanisms contribute to ESCC cell migration [62].

#### 3.1.9. EZR-AS1

Ezrin (EZR), a member of the ezrin-radixin-moesin (ERM) cytoskeletal protein family, has been shown to participate in many aspects of cancer metastasis [63]. Zhang et al. suggested that the lncRNA EZR antisense AS1 (EZR-AS1) can induce ESCC migration by upregulating EZR expression. The lncRNA EZR-AS1 can complex with RNA polymerase 2 and recruit SET and MYND domain containing 3 (SMYD3) to the SMYD3 binding site within the GC-rich region downstream of the EZR promoter, inducing EZR transcription to enhance ESCC cell migration [64].

#### 3.1.10. FTH1P3

The lncRNA ferritin heavy chain 1 pseudogene 3 (FTH1P3) has been reported to promote tumor progression in colorectal cancer [65], cervical cancer [66], and NSCLC [67]. Yang et al. reported that it also functions as an oncogene in ESCC. FTH1P3 was upregulated in ESCC, and silencing FTH1P3 inhibited the migration and invasion of ESCC cells in vitro. Sp1 and NF-κB are both transcription factors involved in cancer metastasis [68], and both are highly expressed in ESCC tissues and cells. Moreover, silencing of FTH1P3 led to the downregulation of Sp1 and NF-kB expression, revealing that FTH1P3 regulates ESCC metastasis by upregulating the SP1/NF-kB pathway [69].

### 3.2. LncRNAs That Function as EC Metastasis Suppressors

#### 3.2.1. MEG3

The lncRNA maternally expressed gene 3 (MEG3) is widely expressed in many normal tissues and has been indicated to be a tumor suppressor [70]. Dong and associates demonstrated that MEG3 was significantly downregulated in EC cells. Treatment of EC cells with the DNA methyltransferase inhibitor 5-AZA-2’-deoxycytidine (5-Aza-dC) induced upregulation of MEG3, which suggested that promoter hypermethylation of MEG3 represents one of the mechanisms that decreases its expression in ESCC tissues. On the other hand, MEG3 may sponge miR-9 to upregulate the expression of E-cadherin and FOXO1, thereby affecting EMT and metastasis [71]. Phosphoserine aminotransferase 1 (PSAT1) has been found to promote the phosphorylation of glycogen synthase kinase-3 beta (GSK-3β) and activate the GSK-3β/Snail pathway, leading to EMT in ESCC [72]. Li et al. suggested that the lncRNA MEG3 can negatively regulate PSAT1 expression and thus inhibit EMT. However, the mechanism by which MEG3 downregulates PSAT1 and EMT still needs further investigation [73].

#### 3.2.2. GAS5

The lncRNA growth-arrest-specific transcript 5 (GAS5) is suppressed in diverse cancers, such as ovarian cancer [74], cervical cancer [75] and gastric cancer [76], indicating that GAS5 may play a tumor-suppressive role. KE et al. suggested that GAS5 is expressed at low levels in ESCC tissues and cells and that the overexpression of GAS5 inhibits ESCC cell invasion and migration by regulating the expression of EMT-associated proteins [77]. Wang et al. demonstrated that the expression of the lncRNA GAS5 in serum decreases as the primary tumor stage increases, and this lncRNA could be a potential diagnostic and prognostic marker. Moreover, they indicated that GAS5 inhibits ESCC migration by inactivating the PI3K/AKT/mTOR pathway [78]. However, in public datasets, Huang et al. suggested that GAS5 is upregulated in ESCC tissues compared with normal tissues but still acts as a tumor suppressor. These authors further indicated that GAS5 was upregulated by interferon (IFN) via the JAK-STAT pathway and that GAS5 can also positively regulate IFN responses. The interaction between GAS5 and the IFN pathway leads to the inhibition of ESCC cell migration and invasion and thus may be a promising therapeutic target in the clinic [79].

#### 3.2.3. BDNF-AS

The lncRNA brain-derived neurotrophic factor antisense (BDNF-AS) was previously found to act as a tumor suppressor in multiple types of cancer including EC [80,81,82,83]. Zhao et al. demonstrated that BDNF-AS expression was significantly decreased in both EC cell lines and tissues and that overexpression of BDNF-AS can suppress the migration, invasion, and EMT of EC cells. Mechanistically, BDNF-AS acts as a ceRNA to sponge miR-214. However, the downstream targets and pathways involved need to be further studied [84].

Cancer metastasis is the main cause of cancer-related death, and studies have shown that lncRNAs serve as double-edged swords in cancer metastasis through diverse mechanisms. Summarizing the mechanism of lncRNAs in EC metastasis might help develop promising diagnostic and prognostic tools and even for developing more effective therapeutics for EC (Table 1).

**Table 1:** The role of lncRNAs in the modulation of EC metastasis.

LncRNA	Mechanism	Location	Reference
	Promotion		
H19	Promotes the STAT3/EZH2/β-catenin axis	11p15.5	[27]
CASC9	Recruits CBP to promoter of LAMC2 and induces LAMC2 transcription, subsequently activating FAK-PI3K/Akt pathways	8q21.11	[30]
MALAT1	Sponges miR-1-3p and activates CORO1C/TPM3 pathway; regulates the Ezh2-Notch1 pathway	11q13	[35,36]
PVT1	Binds to miR-145 and upregulate FSCN1; binds to miR-128 and increase ZEB1 expression	8q24	[43,44]
HOTAIR	Sponges miR-148a and promotes the expression of Snail2; downregulates WIF-1 expression by inducing H3K27 trimethylation in the promoter region and then promoting the Wnt/β-catenin pathway	12q13.13	[46,47]
HOTTIP	Recruits WDR5 and then activates HOXA13 gene transcription; sponges miR-30b and is regulated by miR-30b in the nucleus via an Ago2	7p15.2	[49,85]
LUCAT1	Activates DNMT1 to inhibit the expression of tumor suppressor genes (GADD45G and SFRP2)	5q14.3	[56,86]
CCAT1	Mediates histone methylation and thus represses the expression of tumor suppressor gene SPRY4 in nucleus; competes for miR-7 and then mediates the posttranscriptional regulation of HOXB13 in cytoplasm	8q24.21	[62]
EZR-AS1	Forms a complex with RNA polymerase 2; recruits SMYD3 to the promoter of downstream EZR and promotes EZR transcription	6q25.3	[64]
FTH1P3	Promotes the SP1/NF-κB pathway	2p23.3	[69]
Suppression			
MEG3	Sponges miR-9 to regulate the expression of E-cadherin and FOXO1; downregulates expression of PSAT1	14q32.2	[71,73,87]
GAS5	Inactivates the PI3K/AKT/mTOR pathway; interacts with interferon	1q25	[78,79]
BDNF-AS	Sponges miR-214	11p14.1	[80,84]

## 4. LncRNAs Are Involved in EC Therapy Resistance

**Figure 2:**
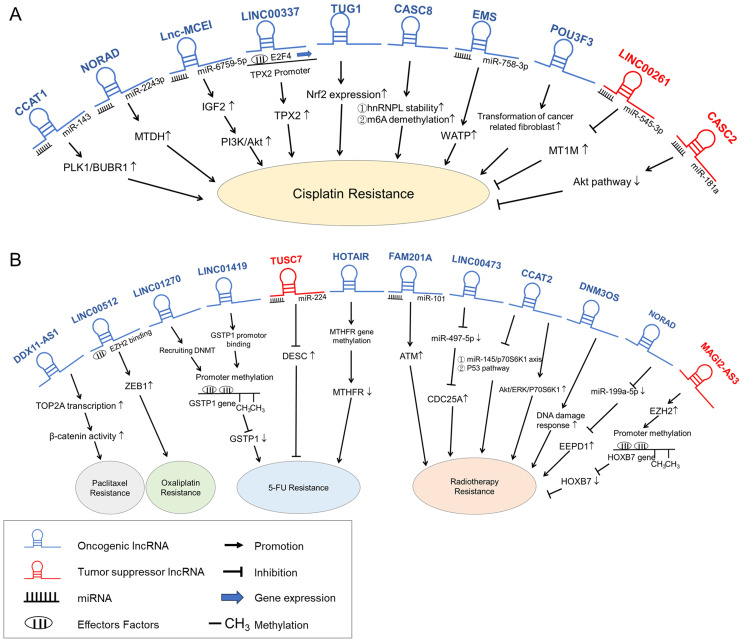
Long noncoding RNA (lncRNA)-controlled pathways in chemoresistance/radiotherapy resistance in esophageal carcinoma cells (ECC). (**A**). These lncRNAs confer cisplatin resistance to ECC cells. (**B**). In addition to cisplatin, these lncRNAs confer resistance to clinical chemotherapeutics including paclitaxel, oxaliplatin, and 5-fluorouracil (5-FU), and these lncRNAs confer radioresistance to patients with ECC. Oncogenic lncRNAs (blue) promote chemoresistance and radiotherapy resistance, while tumor suppressor lncRNAs (red) attenuate chemoresistance and radiotherapy resistance in ECC.

The most suitable treatments for EC differ depending on the tumor stage or features of the patient features. Endoscopic and surgical treatments are used for those with early-stage tumors, while systemic regimens, including definitive chemoradiotherapy, are used to treat patients with locally advanced tumors or those who are not eligible for surgical treatment [88]. However, in addition to metastasis, resistance to anticancer drugs is another clinical obstacle limiting the effectiveness of therapeutic approaches such as chemo-therapy and radiation therapy in cancer patients. Therefore, to improve the poor prognosis of patients with EC, solutions for overcoming drug resistance must be explored. Recently, accumulating evidence has suggested that lncRNAs play critical roles in promoting resistance and decreasing sensitivity to therapeutic drugs in multiple cancers, including EC; thus, they have drawn increasing amounts of attention in the field of cancer (Figure 2 and Table 2).

### 4.1. Chemoresistance

#### 4.1.1. Cisplatin

Cisplatin, or cis-dichlorodiammine-platinum(II), is a representative platinum-based compound that has been used to treat a wide spectrum of solid neoplasms. It interacts with purine bases to inhibit DNA repair and cause DNA damage, thereby facilitating cancer cell apoptosis [89]. Once cisplatin-induced DNA adducts result in DNA lesions, nucleotide excision repair (NER) is the pathway involved mainly in repairing DNA damage [90]. Notably, inhibition of NER effectively improved chemosensitivity to cisplatin as tumor cells with enhanced DNA repair capacity exhibited increased therapeutic resistance [91]. Therefore, targeting the DNA repair machinery seems to be a potential strategy for sensitizing tumor cells to cisplatin. Nevertheless, the involvement of lncRNAs in chemoresistance through the modulation of DNA damage repair has been relatively less reported and warrants further investigation.

Cisplatin-based chemotherapy regimens are still the foremost treatment option for patients diagnosed with EC [92]. Although cisplatin improves EC prognosis, the development of chemoresistance to cisplatin is a common cause of treatment failure [93].

LncRNAs can promote EC resistance to cisplatin by functioning as ceRNAs. The lncRNA CCAT1 contributes to ESCC chemoresistance and facilitates ESCC metastasis. Hu et al. reported that CCAT1 could downregulate miR-143 and thus indirectly increase the expression of polo-like kinase 1 (PLK1) and budding uninhibited by benzimidazole-related 1 (BUBR1), which are associated with the cell cycle. Taken together, these findings indicate that high expression of CCAT-1 facilitates ESCC resistance to cisplatin through the miR-143/PLK1/BUBR1 signaling axis [94]. Noncoding RNA activated by DNA damage (NORAD) was shown to promote multiple types of cancer [95,96], including EC. Jia et al. demonstrated that high NORAD expression was related to poorer prognosis in ESCC patients treated with cisplatin-based therapy, revealing that NORAD overexpression could contribute to cisplatin resistance in ESCC cells. Regarding the underlying mechanism, NORAD can act as a ceRNA to sponge miR-224-3p, thereby upregulating metadherin (MTDH). MTDH was found to participate in multiple pathways, such as the PI3K/Akt and MAPK pathways, and to play important roles in carcinogenesis [97]. In conclusion, the NORAD/miR-224-3p/MTDH axis facilitates Cis-diamminedichloro-platinum (CDDP) resistance by promoting the nuclear accumulation of β-catenin [98]. The roles of lnc-MCEI (a lncRNA that mediates the chemosensitivity of ESCC by regulating IGF2), a lncRNA that mediates the chemosensitivity of ESCC, were first described by Liu and associates. Liu et al. reported that lnc-MCEI was upregulated in ESCC tissues and that knockdown of lnc-MCEI increased the sensitivity of ESCC cells to cisplatin. Mechanistically, the lncRNA lnc-MCEI acts as a ceRNA by sponging miR-6759-5p, thereby inducing the insulin-like growth factor 2 (IGF2)/PI3K/AKT axis. Thus, the MCEI facilitates the chemoresistance of ESCC through the miR-6759-5p/IGF2/PI3K/AKT axis [99].

Some lncRNAs can facilitate EC resistance to cisplatin by interacting with proteins. Yang et al. suggested that LINC00337 is involved in EC progression and chemoresistance. LINC00337 expression is elevated in ESCC tissues and cells compared with normal controls, and silencing LINC00337 increased chemosensitivity to cisplatin. Mechanistically, LINC00337 recruits E2F transcription factor 4 (E2F4) to binding sites within the promotor of the targeting protein for Xenopus kinesin-like protein 2 (TPX2), which was previously found to be related to cell proliferation and a poor prognosis in ESCC patients [100]. Subsequently, TPX2 transcription is promoted, thereby increasing EC cell chemoresistance to cisplatin [101]. Jiang and associates demonstrated that taurine upregulated gene 1 (TUG1) is upregulated in ESCC, especially in tumors resistant to chemotherapy [102]. Zhang et al. further clarified the underlying mechanism related to this phenotype: TUG1 increases nuclear factor erythroid 2-related factor 2 (Nrf2) protein expression by directly binding to the Nrf2 protein. Nrf2 was previously demonstrated to be a key contributor to cisplatin resistance in ovarian cancer [103] and ESCC cells [104]. LncRNA cancer susceptibility candidate 8 (CASC8) contributes to the progression of multiple types of cancer [105,106], and Wu et al. demonstrated the mechanisms of CASC8 upregulation as well as its effect on chemoresistance in ESCC. These authors indicated that AlkB homolog 5 (ALKBH5), an RNA demethylase suppressed the m6A modification of CASC8, which resulted in increased CASC8 levels in ESCC through the stabilization of the CASC8 transcript. However, CASC8 decreases heterogeneous nuclear ribonucleoprotein L (hnRNPL) polyubiquitination and proteasomal degradation, leading to the stabilization and upregulation of hnRNPL protein expression. Taken together, these results indicate that ALKBH5-mediated m6A modification increases CASC8 levels in ESCC and that overexpression of CASC8 facilitates ESCC progression and chemoresistance to cisplatin by interacting with hnRNPL [107].

Interestingly, lncRNAs can also play a role in hypoxia-mediated drug resistance in EC cells, and the lncRNA E2F1 messenger RNA stabilizing factor (EMS) is a salient example of this phenomenon. Zhu et al. suggested that EMS is expressed at greater levels in EC tissue than in adjacent normal tissues and can be upregulated by hypoxia. EMS can sponge miRNA-758-3p and downregulate its expression. Wilms tumor 1-associated protein (WTAP), which has been revealed to be a prognostic marker of several cancers (e.g., nasopharyngeal carcinoma [108] and bladder cancer [109]), serves as the downstream target of miRNA-758-3p. Its overexpression significantly contributes to chemoresistance to cisplatin [110].

On the other hand, several lncRNAs, including LINC00261, increase ESCC sensitivity to cisplatin. Wang et al. reported that LINC00261 expression was significantly lower in ESCC tissues and cells than in normal controls and that LINC00261 could negatively regulate miR-545-3p by sponging. Subsequently, metallothionein-1 M (MT1M), which was previously identified as a tumor suppressor [111], is targeted by miR-545-3p and is thus downregulated in ESCC tissues and cells, leading to resistance to cisplatin [112]. Moreover, in ESCC cells, CASC2 is expressed at low levels. Zhu et al. demonstrated that overexpression of CASC2 in ESCC cells not only promoted DNA damage but also increased sensitivity to cisplatin. CASC2 can directly interact with miR-181a and negatively regulate its expression, thereby suppressing the Akt pathway, which plays a role in multiple cellular processes, such as cell apoptosis and proliferation. [113]. Thus, downregulation of CASC2 in ESCC decreases the antitumor activity of cisplatin by activating the miR-181a/Akt axis [114].

Cancer-associated fibroblasts (CAFs) are a major component of the tumor microenvironment and have multiple functions. They can secrete several factors (e.g., chemokines, cytokines, and growth factors) that degrade their surrounding ECM to support and maintain tumor growth. They also maintain tumor-cell-specific metabolism [115]. Intriguingly, the lncRNA POU3F3 (also known as LINC01158) can promote ESCC resistance through a CAF-associated mechanism. Tong et al. demonstrated that POU3F3 facilitates cisplatin resistance in ESCC by transforming normal fibroblasts (NFs) into cancer-associated fibroblasts (CAFs). Mechanistically, the lncRNA POU3F3 can be packaged into tumor-secreted exosomes and transferred from ESCC cells to NFs, leading to the activation of NFs. Subsequently, activated fibroblasts secrete interleukin 6 (IL-6), which participates in the communication between stromal cells and cancer cells in the tumor microenvironment [116]. Through this mechanism, POU3F3 facilitates ESCC resistance to cisplatin and contributes to a poor prognosis [117].

#### 4.1.2. Fluorouracil (5-FU)

5-FU is widely used in cancer treatment, and it exerts its anticancer effects by inhibiting thymidylate synthase (TS) or by incorporating its metabolites into RNA and DNA [118]. Cisplatin combined with a continuous infusion of 5-FU has been identified as a standard treatment for patients with ESCC or EAC [119], but the development of resistance to 5-FU is common.

Zhang et al. demonstrated that depletion of the lncRNA HOTAIR facilitates the chemosensitivity of EC cell lines to 5-FU. They also found that the methylation level of CpG islands in the promoter region of the methylenetetrahydrofolate reductase (MTHFR) gene was greater in EC tissues than in adjacent normal tissues. Taken together, these results indicate that HOTAIR can repress MTHFR expression by upregulating MTHFR methylation, thereby promoting the chemoresistance of EC cells to 5-FU [120]. In addition, Li et al. suggested that the lncRNA LINC01270 not only promotes EC progression but also contributes to EC cell chemoresistance to 5-FU. LINC01270 is overexpressed in EC tissues, while the expression of GSTP1, which was previously found to be related to drug resistance in EC [121], is low. Mechanistically, they found that LINC01270 can recruit the DNA methyltransferases DNMT1, DNMT3A and DNMT3B to increase the methylation level of glutathione S-transferase P1 (GSTP1), thereby inhibiting the expression of GSTP1. GSTP1 plays diverse roles in the progression of different cancers. For example, GSTP1 was found to serve as an oncogene that suppresses cell apoptosis in head and neck squamous cell carcinoma [122], while another study showed that inhibition of GSTP1 expression promoted EC chemoresistance to 5-FU [123]. LINC01419 also promotes 5-FU resistance through methylation of GSTP1. High expression of LINC01419 has been shown to promote the progression of hepatocellular carcinoma and lung adenocarcinoma [124,125,126]. Chen et al. demonstrated that LINC01419 is highly expressed in ESCC tissues and can bind to the promoter region of the GSTP1 gene, thereby mediating DNA methylation of GSTP1. The hypermethylation of GSTP1 leads to the promotion of ESCC progression and chemoresistance to 5-FU therapy [127].

In contrast, several lncRNAs, including tumor suppressor candidate 7 (TUSC7), can suppress 5-FU resistance. Chang et al. reported that TUSC7 expression was downregulated in ESCC tissues and cells and that TUSC7 could negatively regulate the expression of miR-224. As a downstream target of miR-224, differentially expressed in squamous cell carcinoma 1 (DESC1) is downregulated in ESCC; it attenuates the chemoresistance of ESCC cells to 5-FU or cisplatin by downregulating the EGFR/AKT pathway [128].

#### 4.1.3. Paclitaxel

Paclitaxel is a representative member of the taxane family and exerts its antitumor effects by forming abnormal spindles, which results in chromosome missegregation and the production of aberrant aneuploid daughter cells [129]. Paclitaxel, alone or in combination with cisplatin, has been demonstrated to be an effective first-line chemotherapy for treating EC [130,131], but the development of resistance to paclitaxel compromises its treatment efficacy. Zhang et al. suggested that the lncRNAs DDX11 antisense RNA 1 (DDX11-AS1), topoisomerase II alpha (TOP2A) and TATA-box binding protein-associated factor 1 (TAF1) are upregulated in EC and that the latter two genes contribute to chemoresistance to paclitaxel. Mechanistically, DDX11-AS1 facilitates the transcription of TOP2A by binding to and upregulating the transcription factor TAF1, thereby promoting TOP2A expression. TOP2A increases the activity of β-catenin and promotes its translocation into the nucleus, resulting in EC cell chemoresistance to paclitaxel [132].

#### 4.1.4. Oxaliplatin

Oxaliplatin is a member of the platinum complex that is used for chemotherapy. It exerts antitumor effects by binding to nucleophilic molecules (mainly DNA) and interfering with DNA replication and transcription [133]. Oxaliplatin has become a promising chemotherapeutic agent for ESCC [134], but resistance to this agent is an obstacle in treating this disease [135]. The lncRNA LINC00152 was reported to confer oxaliplatin resistance in colon cancer [136], and it was also recently indicated to be associated with oxaliplatin resistance in EC. Zhang et al. demonstrated that LINC00152 is highly expressed in EC tissues and cells and can bind to the PRC2 protein complex (EZH2). In addition to its role in metastasis, ZEB1 was also demonstrated to regulate drug resistance through multiple different mechanisms [137]. In the present study, the transcriptional inhibition of ZEB1 was attenuated by EZH2 binding, and the overexpression of ZEB1 led to EC cell resistance to oxaliplatin [138].

### 4.2. Radioresistance

Radiotherapy combined with chemotherapy results in a better prognosis than surgery or chemotherapy alone for EC patients [139]. However, some patients develop radiotherapy resistance [140], which is a major obstacle to improving EC treatment efficacy.

Multiple oncogenic lncRNAs can promote radioresistance in patients with EC. The major lesion induced by ionizing radiation (IR) is considered a double-strand break (DSB), which is a break in the phosphodiester backbone of both DNA strands [141]. Nonhomologous end joining (NHEJ) and homologous recombination (HR) are two core mechanisms for DSB repair [142]. Accumulating studies have revealed that enhanced DNA repair machinery can contribute to resistance to IR [143,144]. For instance, IR exposure facilitates the phosphorylation of phosphatase and tensin homolog (PTEN), which subsequently enables the recruitment of RAD51 to augment DNA repair [145]. In addition, high expression of X-ray repair cross complement 3 (XRCC3, a RAD51 paralog) indicated resistance to chemoradiotherapy and poor survival in ESCC patients [144]. Chen et al. suggested that the expression of the lncRNA family with sequence similarity 201-member A (FAM201A) was upregulated in ESCC tissues and related to radiosensitivity. ESCC patients with high FAM201A expression have a poorer short-term response to radiotherapy and a shorter survival time than those with low FAM201A expression. The authors demonstrated that, mechanistically, FAM201A directly interacts with miR-101 as a sponge and negatively regulates miR-101 expression. Ataxia telangiectasia mutated (ATM) is an important repair protein that participates in HR repair. In addition, ATM and mTOR serve as downstream targets of miR-101 and are upregulated when miR-101 is decreased, leading to ESCC radioresistance [146]. Liu et al. suggested that in ESCC cells exposed to X-ray radiation, LINC00473 was upregulated, while miR-497-5p was downregulated. These authors further elucidated that LINC00473 overexpression can downregulate the expression of miR-497-5 and subsequently increase the protein expression of cell division cycle 25A (CDC25A). CDC25A is a significant regulator of the cell cycle, and its high expression contributes to ESCC radioresistance [147].

In addition to its role in chemoresistance, the lncRNA NORAD is also correlated with radioresistance in ESCC. Sun et al. suggested that radiotherapy promotes NORAD expression by increasing H3K4me2 levels. Overexpression of NORAD in the cytoplasm delayed pri-miR-199a1 processing by competitively binding to Pumilio RNA-Binding Family Member 1 (PUM1), thereby inhibiting miR-199a-5p maturation. Conversely, miR-199a-5p is markedly upregulated in sh-NORAD exosomes, and NORAD knockdown facilitates the transfer of exosomal miR-199a-5p into radioresistant cells. Subsequently, endonuclease/exonuclease/phosphatase family domain containing 1 (EEPD1), the target protein of miR-199a-5p, was downregulated, increasing sensitivity to radiotherapy by inhibiting DNA damage repair. In conclusion, NORAD contributes to ESCC radioresistance [148].

The lncRNA colon-cancer-associated transcript 2 (CCAT2) was first identified in colorectal cancer, and its upregulated expression in various types of cancer has been reported to promote tumor progression [149,150,151]. Wang et al. revealed the role of CCAT2 in radiotherapy resistance in EC cells. They found that CCAT2 expression was significantly greater in EC cells and tissues. More intriguingly, the CCAT2 expression level was also greater in patients who had poorer overall survival after radiotherapy treatment, and CCAT2 knockdown enhanced X-ray-induced apoptosis in EC cells, indicating that CCAT2 is involved in radiotherapy resistance in EC. Mechanistically, CCAT2 inhibits miR-145 expression, thereby downregulating the expression of the ribosomal protein S6 kinase beta-1 (p70S6K1), which is a significant downstream kinase of the apoptosis-associated Akt/ERK pathway. On the other hand, they found that CCAT2 not only inhibits the phosphorylation of Akt, ERK and p70S6K1 but also negatively regulates the expression of the p53, p21 and c-Myc proteins in EC cells. Moreover, p53 was demonstrated to increase p21 expression, thereby inhibiting the cell cycle [152], and c-Myc also regulates p21 activity [153]. In summary, CCAT2 promotes the radioresistance of EC cells by inhibiting apoptosis through the repression of the miR-145/p70S6K1 pathway, activation of the Akt/ERK/p70S6K1 pathway and suppression of the p53 pathway [154].

Moreover, some lncRNAs act as tumor suppressors to increase radiosensitivity. According to Cheng and associates, the homeobox protein Hox-B7 (HOXB7) is highly expressed in EC tissues, while the lncRNA MAGI2-AS3 is expressed at low levels. They also demonstrated that overexpression of HOXB7 increased the resistance of EC cells to radiotherapy. Regarding the underlying mechanism, MAGI2-AS3 can recruit EZH2 to the promoter of HOXB7, leading to enrichment of histone H3 lysine 27 trimethylation (H3K27me3) in the HOXB7 promoter region and suppression of HOXB7 expression. Taken together, these findings indicate that MAGI2-AS3 negatively regulates HOXB7 expression, thereby increasing EC sensitivity to radiotherapy [155].

Studies have focused on the correlation between CAFs and lncRNAs related to EC radioresistance. Zhang et al. reported that the lncRNA DNM3OS is highly expressed in ESCC cells and tissues and further suggested that CAFs induce the transcription of DNM3OS by activating the platelet-derived growth factor-β (PDGF-β)/platelet-derived growth factor receptor β (PDGFR-β)/forkhead box protein O1 (FOXO1) pathway. When it is upregulated, DNM3OS can contribute to the enhancement of radioresistance in ESCC by attenuating the DNA damage response [156].

Neoadjuvant therapy, including chemotherapy plus radiotherapy, is thought to be an effective treatment for advanced EC. Currently, therapeutic resistance remains a major obstacle to achieving good clinical responses in patients with EC, and lncRNAs have been found to participate in this process. Elucidating how lncRNAs regulate therapeutic resistance could lead to substantial progress in treating EC to improve patient prognosis (Table 2).

**Table 2:** The role of lncRNAs in modulating chemoresistance and radioresistance in EC cells.

LncRNA	Location	Therapeutic Strategy	Effect on Resistance	Mechanism	Reference
CCAT1	8q24.21 [157]	Cisplatin	Promotion	Sponges miR-143 and then increases the expression of PLK1 and BUBR1	[94]
NORAD	20q11.23 [158]	Cisplatin	Promotion	Sponges miR-2243p and then upregulates MTDH	[98]
		Radiotherapy	Promotion	Inhibits miR-199a-5p maturation and then upregulates EEPD1	[148]
Lnc-MCEI	1q32.1	Cisplatin	Promotion	Sponges miR-6759-5p, thereby upregulates IGF2 and inhibits the PI3K/AKT axis	[99]
LINC00337	1p36.31 [159]	Cisplatin	Promotion	Recruits E2F4 to the promoter of TPX2 and increases TPX2 transcription	[101]
TUG1	22q12 [160]	Cisplatin	Promotion	Binds to Nrf2 to increase its expression	[104]
CASC8	8q24.21 [161]	Cisplatin	Promotion	Is upregulated with ALKBH5-mediated m6A demethylation; interacts with hnRNPL and stabilizes its protein expression	[107]
EMS	7q22.1 [162]	Cisplatin	Promotion	Sponges miR-758-3p and induces WTAP expression	[110]
POU3F3	2q12.1 [163]	Cisplatin	Promotion	Transforms normal fibroblasts into cancer-related fibroblasts	[117]
LINC00261	20 [164]	Cisplatin	Attenuation	Sponges miR-545-3p and upregulates MT1M expression	[112]
CASC2	10q26 [165]	Cisplatin	Attenuation	Downregulates miR-181a expression, and thereby suppresses the Akt pathway	[114]
HOTAIR	12q13.13 [166]	5-FU	Promotion	Represses MTHFR expression by promoting MTHFR methylation	[120]
LINC01270	20q13.13 [127]	5-FU	Promotion	Increases the methylation level of GSTP1 promoter by recruiting DNA methyltransferases, thereby inhibiting GSTP1 expression	[123]
LINC01419	8q21.13 [167]	5-FU	Promotion	Binds to the promoter region of the GSTP1 gene and increases the methylation level of GSTP1	[127]
TUSC7	3q13.31 [168]	5-FU	Attenuation	Sponges miR-224 and then upregulates DESC1 expression	[128]
DDX11-AS1	12p11.21 [169]	Paclitaxel	Promotion	Facilitates TOP2A transcription and then increases the activity of β-catenin	[132]
LINC00512	2p11.2 [170]	Oxaliplatin	Promotion	Binds to EZH2 and then upregulates ZEB1 expression	[138]
FAM201A	9p13.1 [171]	Radiotherapy	Promotion	Sponges miR-101 and then upregulates ATM expression	[146]
LINC00473	6q27 [172]	Radiotherapy	Promotion	Negatively regulates miR-497-5p and increases CDC25A expression	[147]
CCAT2	8q24.21 [173]	Radiotherapy	Promotion	Inhibits apoptosis by suppressing the miR-145/p70S6K1 pathway; activates the Akt/ERK/p70S6K1 pathway; represses the p53 pathway	[154]
MAGI2-AS3	7q21.11 [174]	Radiotherapy	Attenuation	Recruits EZH2 to the promoter region of HOXB7 and then downregulates HOXB7 expression	[155]
DNM3OS	1q24.3 [175]	Radiotherapy	Promotion	Is activated by CAFs and regulates the DNA damage response	[156]

## 5. Discussion

Initially, lncRNAs were viewed merely as transcriptional junk RNAs, but they have been proven to play roles in various organisms and cell types. Accumulating evidence has demonstrated that lncRNAs are involved in the metastasis and drug resistance of EC and can also serve as EC biomarkers and prognostic indicators. For instance, previous studies suggested that lncRNA PVT1 expression is greater in human EC tissues than in adjacent normal tissues [41], and increased expression of HOTAIR was found to be associated with poor overall survival in ESCC patients [176]. Moreover, lncRNAs packaged in exosomes are considered more functional and integral than free lncRNAs because lncRNAs inside exosomes can be protected from RNases; for these reasons, exosomal lncRNAs are viewed as crucial cancer biomarkers [177]. Indeed, growing evidence indicates that exosomal lncRNAs play an important role in the progression of tumors, including EC [117,148,178,179,180]. Exosomes are membrane-bound, nanosized endocytic vesicles containing proteins, lipids and a variety of RNAs [181]. Exosomes are secreted by most cell types, including tumor cells. Recently, tumor-secreted exosomes have been widely studied for their roles in information exchange between cancer cells, particularly during tumor development [182]. Components of exosomes, such as RNAs, miRNAs, proteins, and even metabolites, can influence recipient cells through autocrine and paracrine signaling in many types of cancer [182]. For example, the lncRNA HNF1A-AS1 carried by exosomes upregulates the expression of tuftelin 1 (TUFT1), promoting proliferation and drug resistance in cervical cancer cells [179]. Moreover, the exosomal lncRNA 91H can promote colorectal metastasis by modifying heterogeneous nuclear ribonucleoprotein K (HNRNPK) expression [180]. In addition, as previously mentioned, the lncRNA POU3F3 can also be packaged into tumor-secreted exosomes to regulate ESCC resistance. Thus, because of their significant role in intercellular communication, exosomes are recognized as biomarkers and prognosticators of cancer and can potentially be used as vehicles for clinical gene and drug delivery [183], which largely contributes to EC treatment. However, despite their potential to serve as biomarkers and drug carriers for EC therapeutics, there are still limitations regarding the clinical implications of exosomes. For example, the efficiency of ultracentrifugation, which remains the gold standard for exosome isolation, is low [184]. Moreover, after exosomes are stored at −80 °C and subjected to freeze-thaw cycles, multiple factors, such as cargo concentration, purity, and size, are affected [185]. Thus, additional investigations regarding the utility of exosomes in the clinical setting are still needed.

Metastasis and drug resistance are undoubtedly the two main obstacles to cancer treatment and the main contributors to poor patient survival rates. In this review, we summarize the lncRNAs that are aberrantly expressed in EC and their roles in EC metastasis and drug resistance. With a better understanding of the underlying mechanisms of lncRNAs, we can identify promising biomarkers for diagnosing EC and predicting prognosis. Moreover, with growing interest in the clinical application of lncRNAs, they could become key therapeutic targets soon [186].

## Data Availability

All reports supporting the discussion are available in this paper.
